# Evaluating Solar Wind Forecast Using Magnetic Maps That Include Helioseismic Far-Side Information

**DOI:** 10.1007/s11207-025-02563-4

**Published:** 2025-11-10

**Authors:** Stephan G. Heinemann, Dan Yang, Shaela I. Jones, Jens Pomoell, Eleanna Asvestari, Carl J. Henney, Charles N. Arge, Laurent Gizon

**Affiliations:** 1 Department of Physics, University of Helsinki, P.O. Box 64, 00014 Helsinki, Finland; 2 Institute of Physics, University of Graz, Universitätsplatz 5, 8010 Graz, Austria; 3 Max-Planck-Institut für Sonnensystemforschung, Justus-von-Liebig-Weg 3, 37077 Göttingen, Germany; 4 NASA Goddard Space Flight Center, Heliophysics Science Division, Code 671, Greenbelt, MD 20771 USA; 5 Space Vehicles Directorate, Air Force Research Laboratory, Kirtland AFB, NM 87117 USA; 6 Institut für Astrophysik und Geophysik, Georg-August-Universität Göttingen, 37077 Göttingen, Germany; 7 Center for Astrophysics and Space Science, NYUAD Institute, New York University Abu Dhabi, Abu Dhabi, UAE

**Keywords:** Active regions, Magnetic fields, Solar wind

## Abstract

To model the structure and dynamics of the heliosphere well enough for high-quality forecasting, it is essential to accurately estimate the global solar magnetic field used as inner boundary condition in solar wind models. However, our understanding of the photospheric magnetic field topology is inherently constrained by the limitation of systematically observing the Sun from only one vantage point, Earth. To address this challenge, we introduce global magnetic field maps that assimilate far-side active regions derived from helioseismology into solar wind modeling. Through a comparative analysis between the combined surface flux transport and helioseismic Far-side Active Region Model (FARM) and the base Surface Flux Transport Model without far-side active regions (SFTM), we assess the feasibility and efficacy of incorporating helioseismic far-side information in space weather forecasting. We are employing the Wang-Sheeley-Arge solar wind (WSA) model for statistical evaluation and leveraging the EUropean Heliospheric FOrecasting Information Asset (EUHFORIA), a three-dimensional heliospheric MHD model, to analyze a case study. Using the WSA model, we show that including far-side magnetic data improves solar wind forecasts for 2013 – 2014 by up to $50\%$ in correlation and $3\%$ in root mean square error and mean absolute error, especially near the Earth and Solar TErrestrial RElations Observatory – Ahead (STEREO-A). Additionally, our 3D modeling shows significant localized differences in heliospheric structure that can be attributed to the presence or absence of active regions in the magnetic maps used as input boundaries. This highlights the importance of including far-side information to more accurately model and predict space weather effects caused by solar wind, solar transients, and geomagnetic disturbances.

## Introduction

The continuous stream of plasma emitted by the Sun, known as the solar wind, forms the foundation of heliospheric science, particularly in understanding solar-terrestrial interactions, i.e., space weather (Schwenn 2006; Temmer 2021). Accurate prediction of solar wind behavior is crucial for the operation and protection of satellites, power grids, communication systems, and crewed missions in low-Earth orbit (Buzulukova and Tsurutani 2022).

The solar wind, which originates from the rapid expansion of the solar corona (Parker 1958; Cranmer and Winebarger 2019), is commonly understood to exhibit a bimodal nature. On the one hand, there is the so-called slow solar wind generated via processes mediated by reconnection (although currently there exists no unanimous consensus regarding its origin; Temmer et al. 2023), such as interchange reconnection in the S-web model (separatrix and quasi-separatrix web; Antiochos et al. 2011) and closed field reconnection as observed in active region cusps, among other possible sources. On the other hand, the fast solar wind component is believed to originate in solar coronal holes (Cranmer 2002, and references therein).

As the solar wind flows outward from the Sun in a nearly radial direction, it stretches the coronal magnetic field. Due to the Sun’s rotation, in the interplanetary space the magnetic field takes on an approximately Archimedean spiral shape, the so-called *Parker* spiral (Parker 1958). Consequently, the interaction between slow and fast solar wind forms stream interaction regions (SIRs) that may evolve into recurrent co-rotating interaction regions (CIRs) (e.g. see Jian et al. 2006; Richardson 2018) that are the major cause of weak to moderate geomagnetic activity at Earth (Farrugia, Burlaga, and Lepping 1997; Alves, Echer, and Gonzalez 2006; Verbanac et al. 2011).

The geoeffectiveness of coronal mass ejections (CMEs) can be influenced by the solar wind structure in the heliosphere (Heinemann et al. 2024). When CMEs interact with the solar wind, particularly when they encounter high-speed solar streams (HSSs) or SIRs/CIRs, they may undergo significant modifications as they journey through the heliosphere. These alterations can cause the embedded flux rope to deform, twist, rotate or be deflected (as observed in studies by Manchester et al. 2004; Riley and Crooker 2004; Yurchyshyn 2008; Isavnin, Vourlidas, and Kilpua 2013; Heinemann et al. 2019). The flux rope may also erode due to reconnection processes (Dasso et al. 2006; Ruffenach et al. 2012, 2015; Lavraud et al. 2014; Wang et al. 2018) or be deflected (Wang and Sheeley 2004; Wang et al. 2014; Kay, Opher, and Evans 2013; Zhuang et al. 2019). These interactions can also lead to increased turbulence within the sheath region (Lugaz et al. 2015; Kilpua et al. 2017). All of these effects collectively alter the initial characteristics of CMEs observed near the Sun, and consequently predicting their arrival times and geoeffectiveness becomes a complex undertaking (Richardson 2018). This highlights the critical role of the ambient solar wind structure in studying and forecasting space weather effects.

Solar wind prediction models rely on diverse data sources, and an important factor in their accuracy is the quality and comprehensiveness of the input data. Different methods incorporate various datasets for forecasting purposes and many solar and heliospheric solar wind models, such as the Wang-Sheeley-Arge (WSA: Arge and Pizzo 2000) model, Enlil (Odstrčil and Pizzo 1999), or the EUropean Heliospheric FOrecasting Information Asset (EUHFORIA: Pomoell and Poedts 2018), require an instantaneous estimate of the global solar (photospheric) magnetic field as a prerequisite for constructing the coronal magnetic field model. However, concurrent magnetic field observations of the entire Sun are not available on a regular basis. The common approach of using synoptic charts as an approximation of the instantaneous photospheric full-Sun magnetic field exhibits a drawback known as the “aging effect” (Heinemann et al. 2021). As synoptic maps can only provide $360^{\circ}$ longitudinal information over the span of one solar rotation (≈ 27 days), some parts are distinctly older than others. Although the inclusion of magnetic field observations from viewpoints away from the Earth-Sun line, for example those captured by the Photospheric and Magnetic Imager (PHI: Solanki et al. 2020) on board the Solar Orbiter (SolO: Müller et al. 2020) are highly beneficial, they are not consistently available.

An alternative approach to mitigate the aging effect involves the utilization of surface flux transport models, that attempt to capture the evolution of the large-scale photospheric magnetic field due to meridional flows and differential rotation, along with magnetic diffusion (e.g. ADAPT:^1^ Arge et al. 2010; AFT:^2^ Upton and Hathaway 2014; OFT:^3^ Caplan et al. 2025). However, these models typically do not account for the emergence of new magnetic flux on the Sun’s far-side, and if so, only stochastically. Consequently, emerging flux and even entirely new active regions are only observed when the Sun rotates into Earth’s field-of-view. This causes “outdated” or inaccurate magnetic maps that are used for modeling the solar wind. As a solution proposed by Arge et al. (2013) and subsequently implemented by Yang et al. (2024), the incorporation of far-side information derived from helioseismology into magnetic charts was considered in the latter study. Using solar oscillations, we can infer spherical symmetric averages of the composition, density, and temperature at different depths inside the Sun. Far-side helioseismology utilizes acoustic waves that make multiple refractive skips between the surface and interior of the Sun, where regions of strong magnetic fields influence the effective travel time for a given acoustic wave. The differences in travel times around the solar near-surface interior make it possible to deduce magnetic information about the far-side of the Sun (Lindsey and Braun 2000; Braun and Lindsey 2001; González Hernández, Hill, and Lindsey 2007; Yang, Gizon, and Barucq 2023). In a recent study, Upton et al. (2024) utilized EUV observations as a proxy for far-side active regions and incorporated them into the AFT model, demonstrating that this approach improves the representation of the far-side magnetic field.

In this study, we evaluate the value and feasibility of including far-side information in magnetic maps for space weather forecasting and heliospheric modeling. We analyze WSA solar wind predictions over a two-year period in the ecliptic plane and then further examine changes in the three-dimensional (3D) heliospheric solar wind structure in a case study, focusing on their implications for ambient solar wind forecasting and CME propagation. Through this analysis, we aim to assess the potential of helioseismology to contribute to the advancement of space weather modeling.

## Data and Methodology

To assess the impact of incorporating helioseismic far-side active regions into global photospheric magnetic maps, we analyze solar wind predictions using the semi-empirical WSA solar wind model (Wang and Sheeley 1990; Arge and Pizzo 2000, also see Appendix A.1) using maps from the combined surface flux transport and helioseismic Far-side Active Region Model (FARM: Yang et al. 2024). We compare these maps to predictions based on maps without far-side data, generated by the baseline Surface Flux Transport Model (SFTM) on which FARM is based on. The dataset, produced by Yang et al. (2024), spans the period from 2010 to 2024, during which data from the Solar Dynamics Observatory (SDO: Pesnell, Thompson, and Chamberlin 2012) is available. The data is provided at a resolution of $1^{\circ}$ in both longitude and latitude, with a daily cadence. Further details on the magnetic maps can be found in Yang et al. (2024).

**Figure 1:**
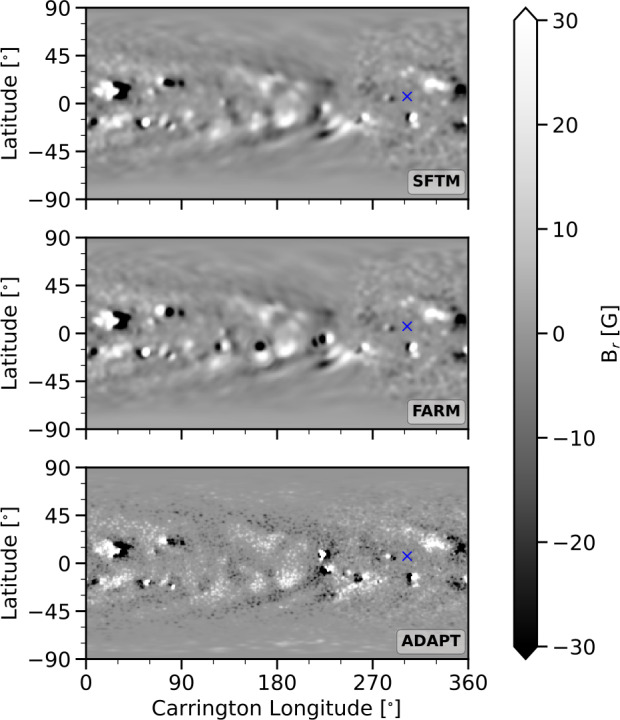
Sample magnetic maps from 2 October 2013 that show the SFTM (before inclusion of far-side active regions), FARM (with far-side active regions included), and ADAPT (reference global magnetic maps without far-side sources). The position of Earth’s longitude is marked by a blue ‘x’.

In addition to the newly developed FARM maps, we also use magnetic maps generated by the well-established Air Force Data Assimilative Photospheric Flux Transport (ADAPT) model (Arge et al. 2010; Hickmann et al. 2015; Barnes et al. 2023), which assimilates data from the Helioseismic and Magnetic Imager (HMI: Schou et al. 2012) on board SDO, to serve as a reference. ADAPT provides 12 realizations for each time, based on variations in supergranular flows. We compute WSA predictions for the full ensemble to estimate the associated uncertainty range. ADAPT maps are used at the same resolution and cadence as FARM and SFTM maps. Figure 1 shows example maps from ADAPT, FARM, and SFTM for 2 October 2013.

We run the WSA model over a two-year period using each of the three types of magnetic maps as input. For this analysis, we selected a time frame between 2013 and 2014, during which both Solar TErrestrial RElations Observatories (STEREO: Kaiser et al. 2008) were operational and positioned at separation angles ranging between $130^{\circ}$ and $170^{\circ}$ from Earth to obtain optimal coverage around the Sun. The WSA model predicts solar wind bulk flow speed at the location of both STEREO spacecraft (A-head and B-ehind) as well as Earth. A more detailed description can be found in the Appendix A.1.

**Figure 2:**
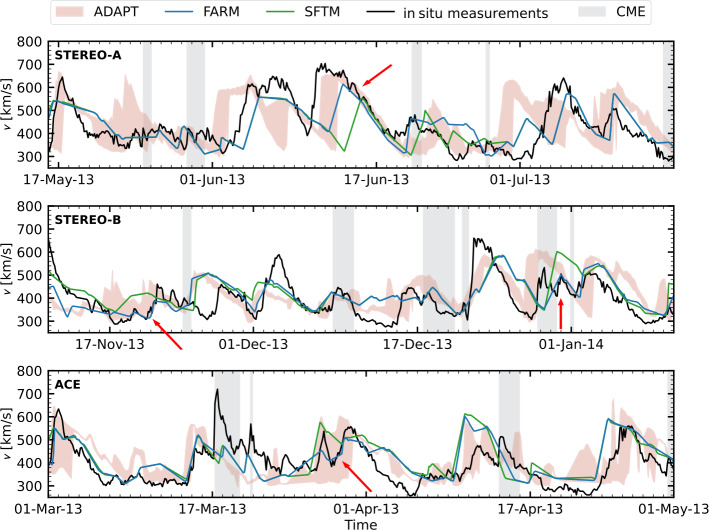
Modeled and observed in situ solar wind velocities are shown for the following time periods: 16 May to 16 July 2013 for STEREO-A, 11 November 2013 to 11 January 2014 for STEREO-B, and 1 March to 1 May 2013 for ACE. The observed in situ velocity is shown in black. Model predictions based on and SFTM maps are represented in blue and green, respectively. The red shaded region indicates the range of solutions from the ADAPT ensemble. Gray shaded areas denote time intervals identified as CMEs in the Richardson and Cane ICME catalog (ACE) or L. Jian’s ICME lists (STEREO-A, -B). Red arrows highlight periods where results visibly outperform SFTM results.

To evaluate the model results, we use solar wind in situ measurements from the Advanced Composition Explorer (ACE: Stone et al. 1998) for Earth’s location. For STEREO, we use plasma data from PLAsma and SupraThermal Ion Composition instrument (PLASTIC: Galvin et al. 2008). For consistency when making comparisons, the observed solar wind data (originally at 1-hour resolution) and the WSA model output (provided on a non-uniform time grid) were both interpolated to a uniform 3-hour cadence. We remove all CME intervals provided in the Richardson and Cane ICME (interplanetary CME) list^4^ (RC-list: Richardson and Cane 2010) from ACE and the respective model data. For the two STEREO spacecraft, we use the ICME list from L. Jian^5^(Jian et al. 2018). A two month segment of the in situ time series, including model results at each location, is shown in Figure 2.

Using both the measured and predicted solar wind data, we conduct a standard classification performance evaluation and time series correlation. We compute the root mean square error (RMSE), the mean absolute error (MAE), the Pearson correlation coefficient as well as a Dynamic Time Warping (DTW) analysis (Samara et al. 2022). DTW is a distance-based similarity measure used to compare two time series that may vary in speed or timing. It identifies an optimal nonlinear alignment between the time series by minimizing the cumulative distance between them. In this context, lower DTW values indicate better agreement between the model and observations, as they reflect smaller discrepancies in both the shape and timing of the profiles. More details are shown in Appendix B.

Table 1 presents the solar wind velocity performance metrics for the three time intervals shown in Figure 2. Across all spacecraft (STEREO-A, STEREO-B, and ACE) and nearly all metrics, Pearson correlation coefficient, RMSE, MAE, and DTW, the runs using FARM as input consistently outperform runs using both SFTM and ADAPT maps. Notably, FARM maps achieve higher correlation values and lower error metrics, indicating better agreement with in situ observations. We further quantify this performance over a longer, two-year period later in the manuscript.

**Table 1:** Solar wind velocity metrics for the time intervals shown in Figure 2.

	FARM	SFTM	ADAPT	FARM	SFTM	ADAPT
	$cc_{\mathrm{Pearson}}$			RMSE [km/s]		
STEREO-A	0.63	0.50	0.31 – 0.54	83	95	97 – 118
STEREO-B	0.52	0.52	0.39 – 0.55	74	79	75 – 86
ACE	0.60	0.56	0.33 – 0.57	75	83	78 – 96
	MAE [km/s]			DTW		
STEREO-A	63	67	71 – 82	5.9	8.3	7.6 – 10.4
STEREO-B	56	61	57 – 67	9.4	9.2	11.3 – 12.9
ACE	59	67	61 – 78	8.1	9.4	9.1 – 12.3

To qualitatively evaluate the FARM magnetic maps for advanced 3D heliospheric modeling, we use the 3D magnetohydrodynamic (MHD) heliospheric model EUHFORIA (Pomoell and Poedts 2018). It is a space weather modeling suite that utilizes a blend of empirical and physics-based methodologies to compute the temporal evolution of the inner heliospheric plasma environment. It comprises two primary components: a coronal model and a heliosphere model with the option to incorporate various configurations of coronal mass ejections. The coronal model generates data-driven solar wind plasma parameters at 0.1 AU through the construction of a magnetic field model representing the large-scale coronal magnetic field, along with the application of empirical relationships to ascertain plasma characteristics like solar wind speed and mass density. These parameters serve as boundary conditions to drive a 3D MHD model of the inner heliosphere, which extends from 21.5 R_⊙_ up to 2 AU. More details regarding the model setup can be found in the Appendix A.2.

## Results

### Statistical Evaluation of WSA Solar Wind Forecast

**Figure 3:**
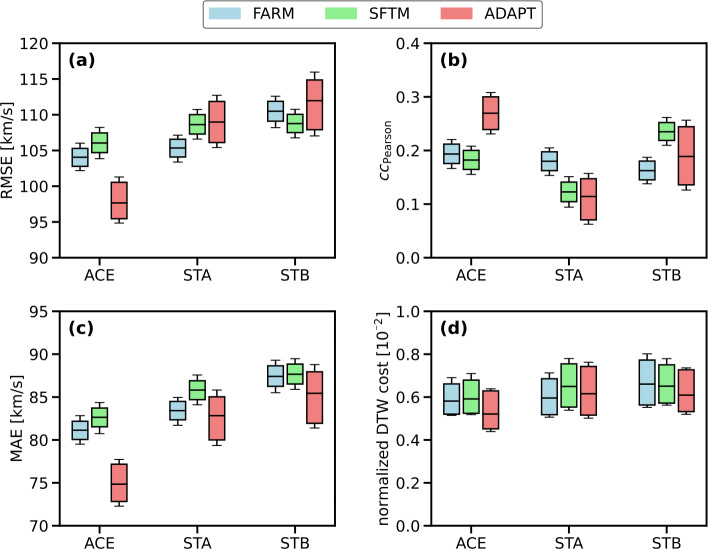
Statistical evaluation of two years of WSA results from FARM, SFTM, and ADAPT maps for the location Earth (ACE), STEREO-A (STA) and STEREO-B (STB) respectively. Panel a and c show the RMSE and MAE between model results and in situ measurements, panel b depicts the Pearson correlation coefficient, and panel d the normalized DTW distance. The bars and error bars represent the $80\%$ and $95\%$ confidence intervals, respectively, while the horizontal bar indicates the median value.

From two years of predicted solar wind data using the WSA model with FARM, SFTM, and ADAPT providing the global photospheric magnetic field maps, we derived the overall agreement between models and observations using the Pearson correlation coefficient ($cc_{\mathrm{Pearson}}$). We found that FARM performs between 6 – 50% better than the base SFTM for STEREO-A and ACE over the full time period, but worse for STEREO-B. The reference maps from ADAPT, which are tuned to work well with WSA, best perform for ACE but are worse than FARM at STEREO-A. This is visualized in Figure 3b.

To assess the agreement between the model results and in situ observations, we calculated the root mean square error (RMSE) and mean absolute error (MAE) of the solar wind speed, as defined in Equations 3 and 4 (in Appendix B). FARM and SFTM show very similar performance in terms of both RMSE and MAE, with FARM achieving values up to $3\%$ lower compared to SFTM. For the STEREO-A and -B spacecraft, FARM outperforms ADAPT in terms of RMSE by up to $3.5\%$, but shows a higher MAE. As expected, ADAPT performs best for ACE. These results are illustrated in Figure 3a,c. Overall, all model results are within $15\%$ of each other, with ADAPT exhibiting the greatest variability due to its 12 different realizations.

Figure 3d shows the normalized DTW distance, revealing that all three models produce similar results across all locations. FARM maps perform equal to or better than SFTM maps for all locations, with FARM performing up to $9\%$ better than SFTM and up to $5\%$ better than ADAPT for STEREO-A. ADAPT performs best for ACE. However, the confidence intervals are large and overlap, making it difficult to draw definitive conclusions about the relative performance of the models. All statistical results are listed in Table 3.

### The 3D Solar Wind Structure

In Section 2, we conducted a statistical evaluation of magnetic maps incorporating far-side active regions using the WSA solar wind model. While this method enables the mapping of the solar wind speed at specific locations in the heliosphere (e.g. Earth, STEREO-A, and -B), it does not facilitate an assessment of the 3D heliospheric structure. To illustrate potential changes in the overall structure, changes that are not only relevant to mapping the background solar wind structure but are also capable of significantly affecting the propagation behavior of solar transients, we utilized the heliospheric MHD model EUHFORIA.

**Figure 4:**
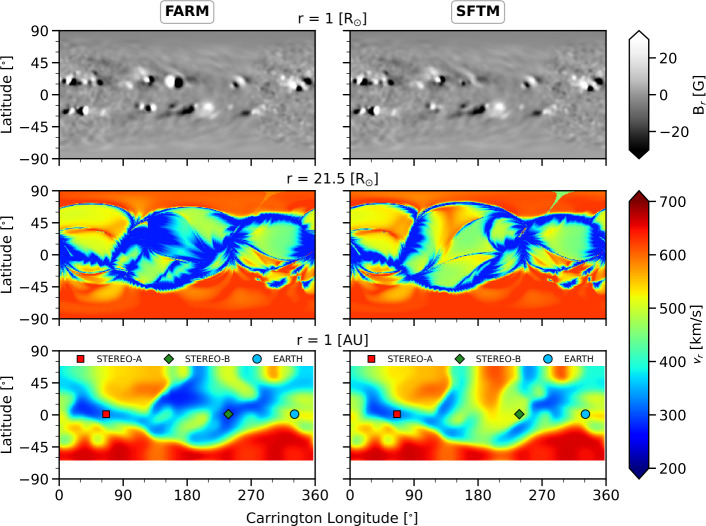
EUHFORIA model results using FARM (left column) and SFTM magnetic maps (right column) from 8 June 2011. From top to bottom the panels depict the photospheric magnetic field that was used as input to the coronal model, the solar wind at 0.1 AU which is the input to the heliospheric model, and the solar wind results at 1 AU. The simulation results correspond to 15 June 2011 23:59 UT. The blue, red, and green dots denote the projected locations of Earth, STEREO-A, and STEREO-B, respectively.

We ran EUHFORIA using a FARM and a SFTM magnetic map from 8 June 2011. This date was selected because it includes multiple far-side active regions, thus exhibiting significant differences in the photospheric far-side magnetic field. The top panels of Figure 4 show the input maps, where FARM features large active regions that are absent from the SFTM map. These discrepancies directly affect the coronal modeling results, as evidenced by differences in the solar wind speed at 0.1 AU (21.5 R_⊙_). The most notable difference appears in the SFTM results, which display a region of fast solar wind around 120^∘^ – 150^∘^ Carrington longitude and approximately $45^{\circ}$ latitude, a feature not present in the FARM results. This suggests that the HSS observed in the SFTM case may be an artefact caused by incomplete magnetic field information, as this structure is absent in the FARM simulation and also not seen at 1 AU.

Due to these far-side differences, the solar wind profiles at 1 AU show significant variation in the Carrington longitudes between 30^∘^ – 150^∘^, while appearing more similar near the Earth-facing (front-side assimilation) region, an expected outcome. Additionally, we find that the solar wind streams encountered by Earth, STEREO-A, and STEREO-B are generally consistent between the two models, although the 3D structure significantly differs in certain regions.

**Figure 5:**
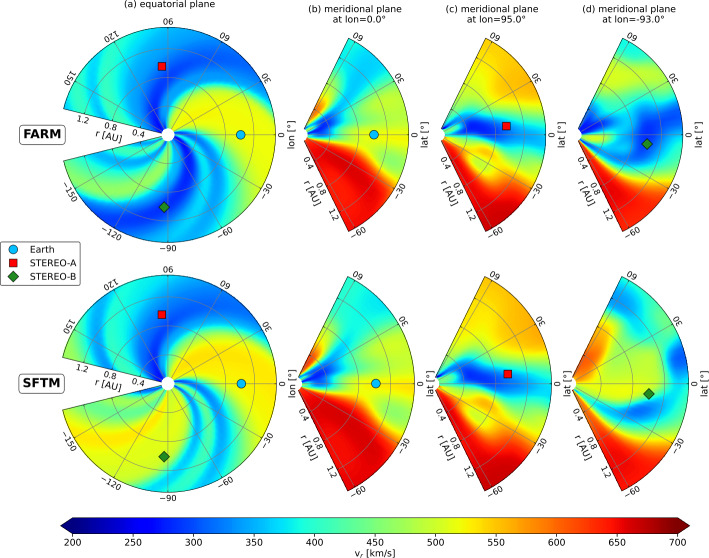
EUHFORIA model results of the same run as Figure 4 but showing the equatorial plane (column a) and the meridional planes at the position of Earth (column b), at STEREO-A (column c), and STEREO-B (column d). The simulation results correspond to 15 June 2011 23:59 UT. FARM results are shown on the top row and SFTM results on the bottom row. The blue, red, and green dots denote the projected locations of Earth, STEREO-A, and STEREO-B, respectively. Note that the longitudes are given in Stonyhurst coordinates for clarity.

To provide a clearer view of the results, Figure 5 presents the equatorial plane along with the meridional planes at the locations of Earth, STEREO-A, and STEREO-B. As already seen in the radial planes (Figure 4), the most significant differences appear near the solar far-side, where FARM clearly shows the absence of an HSS in the northern hemisphere, visible in both the equatorial plane and the meridional plane at the location of STEREO-A. In contrast, the meridional planes at Earth and STEREO-B reveal largely similar modeled solar wind speeds, particularly at the spacecraft locations.

**Figure 6:**
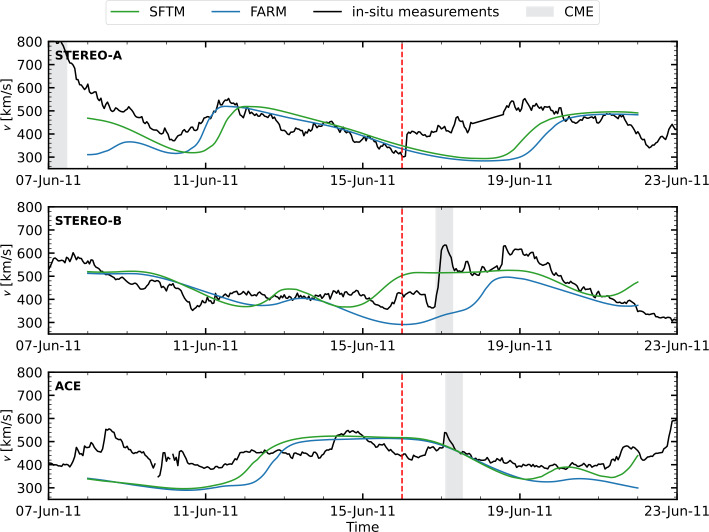
EUHFORIA solar wind predictions for STEREO-A (top panel), STEREO-B (middle panel), and Earth (bottom panel) using FARM (blue line) and the SFTM (green line) results in comparison to the respective in situ measured data (black line). The gray shaded areas denote time intervals identified as CMEs in the Richardson and Cane ICME catalog (ACE) or L. Jian’s ICME lists (STEREO-A, -B). The vertical red line marks 15 June 2011 23:59 UT, the date at which the simulation results are shown in Figures 4 and 5.

This is further confirmed in Figure 6, which shows the EUHFORIA solar wind predictions as a function of time at Earth, STEREO-A, and STEREO-B. While the modeled solar wind from both magnetic maps aligns reasonably well with the in situ measurements, and with each other, for most of the time period, there are some differences, particularly in the STEREO-B results. Here, SFTM predicts an earlier onset and a longer duration of an HSS compared to FARM results. However, due to the presence of a CME in the in situ data during that period, a definitive assessment of whether FARM or SFTM aligns better with observations is not possible—though the results from FARM appear more consistent with the expected solar wind structure.

## Discussion

During periods of enhanced solar activity, the prediction of the solar wind speed is particularly difficult due to the complexity of the solar magnetic field configuration, enhanced by the frequent emergence of active regions out of Earth’s field-of-view. Operative solar wind models generally perform at a long-term correlation of $cc = 0.2$ – 0.5 (Reiss et al. 2020), strongly varying between solar minimum and maximum. Milošić et al. (2023) evaluated the state-of-the-art operative empirical model Empirical Solar Wind Forecast (ESWF) 3.2 to have a correlation of $cc = 0.31$ during solar maximum. All our model runs were found to perform in the range of $cc \approx0.11$ – 0.27. Although over short time periods, the correlation can easily exceed $cc = 0.5$. As expected, FARM and SFTM results are most similar at Earth, as the assimilated data is most accurate. Unexpectedly, FARM does not outperform SFTM at STEREO-B but rather on both STEREO-A and Earth. Used as a reference model, the ADAPT maps perform best for Earth. Here we note again that WSA is tuned for initiation with ADAPT maps and in situ observations at Earth’s position, and therefore expected to perform better than other maps. In addition to comparing model performance, it is important to note the general trend in observational data quality and model accuracy across spacecraft. Performance metrics such as MAE and RMSE are consistently best for ACE, followed by STEREO-A, and then STEREO-B. This ordering aligns with the availability of magnetic field information: ACE benefits from direct Earth-side observations, STA corresponds to regions that have just rotated out of Earth view and therefore has partial, more recent Earth-based magnetic field input, while STB observes far-side regions where the magnetic field information is oldest, except for updates incorporated through FARM. This gradient in data currency and quality naturally influences the model accuracy at each spacecraft location.

MacNeice (2009) and Jian et al. (2015) derived RMSE for solar wind predictions at Earth of 100 – 150 km s^−1^, which we are able to match in our study for all three locations, as well as input magnetic maps. ADAPT performs worst at STEREO-B at an RMSE of 112 km s^−1^ and best at 98 km s^−1^ at Earth. FARM and SFTM perform very similarly across all locations within ± 5 km s^−1^ of each other in the RMSE range of 100 – 110 km s^−1^. This is also in agreement with Milošić et al. (2023) who found a RMSE of $110\text{ km}$ s^−1^.

Samara et al. (2022) established DTW as a reliable method for comparing time series in heliospheric physics. However, in our case, we find a large spread and very similar values across all models and locations, although there is a slight indication of improved performance from the FARM maps compared to SFTM.

Arge et al. (2013) were the first to implement far-side active regions in a flux transport model for solar wind forecasting. By inserting active regions into two Carrington rotations, they achieved a qualitative improvement of the forecast using WSA runs. Their results agree with those derived in this study. Knizhnik et al. (2024) analyzed the AFT-304 model, which incorporates far-side information via STEREO He ii observations, and found that even when far-side active region emergence was clearly inferred, the differences in in situ solar wind predictions were minimal in most cases. Only under specific conditions did the inclusion of this data lead to noticeable differences in the predicted solar wind velocity. This selective impact is similar to our own findings, with overall improvements in ecliptic-plane forecasts remaining generally modest. Similarly, our results support their conclusion that a far-side active region must significantly displace open flux or alter magnetic connectivity in order to meaningfully affect solar wind conditions at observational points. Perri et al. (2024) further support this interpretation, demonstrating that even large far-side active regions may have negligible impact on solar wind conditions at Earth. Using a potential-field source-surface (PFSS) modeling of NOAA 12803 during Carrington rotation (CR) 2240, they found minimal differences in solar wind speed and coronal structure when comparing simulations with and without an inserted far-side bipole.

While the improvement in predicted solar wind speeds within the ecliptic plane is relatively small, substantial differences become evident when considering the 3D structure. Large-scale changes in solar wind structure (such as absent or present HSSs) are possible, as illustrated in Figures 4 and 5. These changes may not directly impact the solar wind within the ecliptic, but they can significantly influence the propagation of CMEs in heliospheric models. CMEs can be notably deformed or deflected by the presence or absence of HSSs, meaning that even variations in solar wind structure outside the ecliptic can substantially affect predictions of geomagnetic storms at Earth or other planets. One mechanism contributing to these off-ecliptic structural differences is the dynamic evolution of coronal holes and active regions. Wang et al. (2024) highlighted that the influence of newly emerged ARs is strongly modulated by their location, polarity, and interaction with preexisting coronal holes. While the total dipole or quadrupole moment may remain stable, interchange reconnection can still locally reshape coronal hole boundaries, causing latitudinal shifts in HSS footprints or temporary modifications to wind speed. This helps explain the cases in our study where FARM and SFTM magnetic maps produce qualitatively different solar wind structures off the ecliptic, even if in-ecliptic differences remain modest.

Generally, we do not expect significant improvements in background solar wind prediction from the inclusion of far-side active regions in magnetic maps over extended periods. Instead, the impact is likely to be more pronounced during shorter, more localized, intervals when the solar far-side evolves differently from the front side, i.e., when the magnetic field structure on the far-side deviates from that assumed by flux transport models or traditional synoptic charts.

Another reason for the relatively modest improvements in ecliptic solar wind predictions is the typical location of active regions away from the solar equator (up to ≈ 35^∘^ latitude depending on the solar cycle; Weber et al. 2023). Larger improvements might be expected near the end of solar maximum, when active regions tend to emerge at lower latitudes, but, during solar minimum, little to no difference between FARM and SFTM results is expected.

It is also worth noting that the inserted far-side active regions are assumed to have zero net magnetic flux. This means that the overall flux balance of the magnetic map remains unchanged, and therefore, minimal large-scale restructuring may occur. Finally, active regions generally do not emerge within large open-field regions, i.e., coronal holes which are the source regions of HSSs (Cranmer 2002; Heinemann et al. 2018, 2020). As a result, cases in which the presence or absence of far-side active regions leads to significant changes in HSSs are likely the exception rather than the rule.

## Summary and Outlook

In this study, we evaluated magnetic maps, which include far-side active regions derived from helioseismic measurements, as input to the WSA solar wind prediction model, as well as to the heliospheric MHD model EUHFORIA.

Our findings can be summarized:

- Both FARM and SFTM align well with state-of-the-art magnetic maps like ADAPT, delivering reliable solar wind forecasts in the ecliptic plane during the tested solar maximum period and serving as viable inputs for the WSA and EUHFORIA models.
- Using the WSA model, we demonstrate that magnetic maps that incorporate far-side information improve long-term solar wind predictions for 2013 – 2014 by up to 6 – 50% in terms of the Pearson correlation coefficient, and by up to $3\%$ for RMSE and MAE, particularly at the locations of Earth and STEREO-A.
- Using advanced 3D modeling, we highlight how changes in the far-side photospheric magnetic field can significantly alter the solar wind structure, potentially enhancing the geoeffectiveness of solar transients.
- While localized and global differences in solar wind structure can be substantial, particularly off the ecliptic, improvements in predicting solar wind speed at Earth remain moderate, reflecting the limitations of current models and the complex influence of far-side magnetic activity.

The evaluation in this study focused solely on the improvement of FARM from the base SFTM due to the inclusion of the far-side active regions, as described in Yang et al. (2024), without deviation from the default model settings. In this article, we show the benefits of the inclusion of far-side information over a standard surface flux transport model. Going forward we will take further steps, for example, optimizing surface flux transport model parameters such as surface flows or diffusion, adjusting the settings of the solar wind model, and even using the methodology of detecting and inserting the far-side active regions detected using helioseismic holography (see Yang, Gizon, and Barucq 2023) in collaboration with more advanced surface flux transport models. We expect that the resultant new global solar magnetic maps will contribute significantly to advancing heliospheric research and improving space weather forecasting.

## Data Availability

Data generated during the current study are available from the corresponding author on reasonable request.

## Appendix A: Model Setup

### A.1 Wang-Sheeley-Arge Model: WSA

The WSA model is composed of three primary stages. First, the PFSS model calculates the inner coronal magnetic field by extrapolating from the photosphere to the source surface (R${\mathrm{SS}}$). This is achieved via a spherical harmonic expansion that matches the observed photospheric flux and imposes a radial magnetic field at R${\mathrm{SS}}$. Next, the solution is extended outward from the Schatten current sheet (SCS) radius (R${\mathrm{SCS}}$) to 5 solar radii using the SCS model. This method simulates the formation of a current sheet and helmet streamer above the magnetic neutral line by temporarily assuming a unipolar radial field and resolving a potential field to preserve open field lines and minimize the energy of the system. Finally, the magnetic field at 5 solar radii is used to estimate the solar wind speed through an empirical relationship. This empirical relationship used to estimate solar wind speed commonly depends on two key parameters: the magnetic flux tube expansion factor ($f_{ \text{exp}}$; e.g., Wang and Sheeley 1990, 1992; Wang, Hawley, and Sheeley 1996) and the angular distance from the coronal hole boundary ($\Theta_{B}$ or DCHB; e.g., Riley, Linker, and Mikić 2001; Riley, Linker, and Arge 2015). The expansion factor $f_{\text{exp}}$ is inversely correlated with solar wind speed and is associated with the modulation of outflow velocities along open magnetic field lines, particularly in wave–turbulence-driven acceleration models. In contrast, $\Theta_{B}$ measures the angular separation between a field line footpoints and the nearest coronal hole boundary, serving as a proxy for the likelihood of magnetic reconnection processes.

Recent studies (Riley et al. 2012; Riley, Linker, and Arge 2015; Wallace et al. 2020) indicate that the traditional inverse relationship between $f_{\text{exp}}$ and solar wind speed breaks down in certain magnetic topologies, such as pseudostreamers. Consequently, modern implementations of the WSA model, including the version used in this study, place greater emphasis on the coronal hole boundary distance parameter ($\Theta_{B}$).

In this study we used R$_{\mathrm{SS}} =2.51$R_⊙_, R$_{ \mathrm{ScS}} =2.49$R_⊙_ with the empirical solar wind relation:

1 $$ V = V_{0} + \frac{V_{m}}{(1+f_{\text{exp}})^{C_{1}}}\, \left(1 - C_{2}\,\text{exp}\left[-\left(\frac{\Theta_{B}}{C_{3}} \right)^{C_{4}}\right]\right)^{C_{5}} $$

where $V_{0}$, $V_{m}$, and $C_{1}$ – $C_{5}$ are parameters that were empirically derived. They are listed in Table 2.

**Table 2:** Parameter values used for solar wind speed predictions in Equation 1.

$V_{0}$	$V_{m}$	$C_{1}$	$C_{2}$	$C_{3}$	$C_{4}$	$C_{5}$
286 m/s	625 m/s	2/9	0.8	1	2	3

### A.2 European Heliospheric FOrecasting Information Asset: EUHFORIA

EUHFORIA is a computational framework designed to simulate large-scale solar wind and magnetic field behavior within the inner heliosphere. Starting from the inner boundary at a heliocentric distance of $r_{H} = 0.1$ au, the evolution of plasma and magnetic structures for $r > r_{H}$ is governed by numerically solving the 3D magnetohydrodynamic (MHD) equations, as described in Pomoell and Poedts (2018).

The solar wind background at $r = r_{H}$ can be initialized using several methods. In this study, a semi-empirical approach, similar to that of the WSA model (refer to A.1), is used. Specifically, the coronal magnetic field from the solar surface up to $r_{H}$ is reconstructed using a two-part system: a PFSS model for the lower corona and a Schatten current sheet (SCS) model for the upper corona. These are implemented using the method of expansion in solid harmonics up to degree l = 140 to solve the Laplace equation, with the PFSS model extending to a source surface radius of 2.6 R_⊙_ and the current sheet model beginning at a lower height of 2.3 R_⊙_. This parameter configuration is a similar approach to that of the WSA model and is known to significantly influence the structure of the extrapolated magnetic field (e.g. McGregor et al. 2008; Asvestari et al. 2019). The numerical grid used includes a radial resolution of approximately $\Delta r \approx0.02$ R_⊙_ for the PFSS and $\Delta r \approx0.15$ R_⊙_ for the SCS domain, with an angular resolution of $1^{\circ}$, matching the input magnetograms.

Magnetic field lines are traced through the coronal model to assess connectivity, and a map of the angular distance $d$ to the closest open field line is generated. The solar wind speed is then assigned using the empirical relation:

2 $$ V = V_{\mathrm{s}} + V_{\mathrm{f}}\frac{1}{(1+f)^{\alpha}} \left( 1 - 0.8e^{(-\frac{d}{w})^{\beta}} \right)^{3} $$

where $V_{\mathrm{f}} = 675\text{ km}/\text{s}$, $V_{\mathrm{s}} = 240\text{ km}/\text{s}$, $\alpha= 0.22$, $\beta= 1.25$, and $w =0.01~\text{rad}$ and $f$ is the flux tube expansion factor, similar to the form used by McGregor et al. (2011).

The MHD simulation is executed over a 10-day period to achieve a quasi-steady-state solar wind configuration (no CMEs are included). The computational domain spans a radial range from 0.1 to 2.0 au and latitudes from $-70^{\circ}$ to $70^{\circ}$, using a grid of $(512, 35, 90)$ cells in radial, latitudinal, and longitudinal directions, respectively.

## Appendix B: Statistics

We calculate the mean absolute error (MAE) and the root mean square error (RMSE) between the modeled and the in situ measured solar wind velocities for the entire 2-year timeseries.

3 $$ \mathrm{MAE} = \frac{1}{n} \sum_{i=1}^{n} |y_{i} - \hat{y}_{i}| $$

4 $$ \mathrm{RMSE} = \sqrt{\frac{1}{n} \sum_{i=1}^{n} (y_{i} - \hat{y}_{i})^{2}} $$

where $n$ represents the number of data points (in the model) or observations, $y_{i}$ is the actual (observed) value for the $i$-th data point and $\hat{y}_{i}$ represents the predicted (modeled) value for the $i$-th data point.

To quantify the similarity between time series from model output and in situ observations, we compute the normalized Dynamic Time Warping (DTW) distance using the dtaidistance Python package. DTW is a time-series comparison algorithm that aligns two sequences by minimizing the cumulative distance between them, allowing for temporal shifts and local stretching or compression. This makes DTW particularly well-suited for comparing solar wind profiles, which may exhibit similar shapes but be offset in time due to variations in propagation speed or arrival time.

Before computing DTW, each time series is standardized by subtracting its mean and scaling to unit variance. This ensures that the comparison emphasizes the shape of the time series rather than their absolute amplitude differences. The DTW distance is then computed between the standardized model and observational series.

To account for differences in time series length, we normalize the DTW distance by the number of data points, yielding a scale-independent similarity metric. In this formulation, a lower normalized DTW value indicates greater similarity between the time series in both pattern and timing, while a higher value reflects larger misalignments or structural differences.

To assess the robustness of the DTW results, we compute confidence intervals by varying the DTW window size, which controls the maximum temporal displacement allowed during alignment. This provides an estimate of the uncertainty associated with the DTW-based similarity metric.

This approach allows us to capture not only amplitude and shape differences, but also timing variations between modeled and observed solar wind features, making DTW a more flexible and informative metric than traditional point-by-point comparisons.

**Table 3:** Solar wind velocity metrics for the full time interval of two years, 2013 and 2014. The values in the brackets show the $95\%$ confidence intervals.

	FARM	SFTM	ADAPT
	$cc_{\mathrm{Pearson}}$		
STEREO-A	0.18 [0.16;0.20]	0.12 [0.10;0.14]	0.11 [0.07;0.15]
STEREO-B	0.16 [0.15;0.18]	0.24 [0.22;0.25]	0.19 [0.14;0.24]
ACE	0.19 [0.18;0.21]	0.18 [0.16;0.20]	0.27 [0.24;0.30]
	RMSE [km/s]		
STEREO-A	105.40 [104.18;106.59]	108.69 [107.36;110.11]	108.96 [106.11;111.82]
STEREO-B	110.41 [109.03;111.76]	108.78 [107.40;110.13]	111.97 [108.09;114.87]
ACE	104.03 [102.73;105.29]	105.93 [104.50;107.31]	97.69 [95.35;100.60]
	MAE [km/s]		
STEREO-A	83.44 [82.32;84.59]	85.86 [84.75;87.01]	82.82 [79.99;85.06]
STEREO-B	87.30 [86.09;88.47]	87.68 [86.51;88.92]	85.44 [82.13;88.04]
ACE	81.13 [80.05;82.14]	82.55 [81.42;83.75]	74.87 [72.81;77.23]
	normalized DTW cost [10^−2^]		
STEREO-A	0.60 [0.51;0.71]	0.65 [0.54;0.78]	0.62 [0.50;0.76]
STEREO-B	0.66 [0.55;0.80]	0.65 [0.56;0.78]	0.61 [0.52;0.74]
ACE	0.58 [0.51;0.69]	0.59 [0.52;0.71]	0.52 [0.44;0.64]

## Footnotes

1 Air Force Data Assimilation Photospheric Flux Transport model.

2 Advective Flux Transport model.

3 Open-source Flux Transport model.

4 https://izw1.caltech.edu/ACE/ASC/DATA/level3/icmetable2.htm.

5 https://stereo-ssc.nascom.nasa.gov/pub/ins_data/impact/level3/.
